# Cholestatic Pruritus Mimicking a Cutaneous Drug Eruption

**DOI:** 10.7759/cureus.49049

**Published:** 2023-11-19

**Authors:** Akhila M Reddy, Maleka Najmi, Palak Parekh

**Affiliations:** 1 Department of Internal Medicine, Texas Tech University Health Sciences Center School of Medicine, Lubbock, USA; 2 Department of Dermatology, Baylor Scott & White Medical Center, Temple, USA

**Keywords:** extrahepatic cholestasis, prurigo nodularis (pn), cholestatic pruritus, cholestasis, pruritus

## Abstract

Pruritus, colloquially known as itch, is a common clinical symptom seen in a variety of dermatological conditions and systemic disorders. Pruritus can broadly be classified into four categories: neuropathic, neurogenic/systemic, psychogenic, and pruritoceptive. Initial categorization depends on anatomical and pathophysiological aspects of presentation and is reflective of underlying etiology. We report a case of an 83-year-old man presenting with generalized pruritus secondary to cholestasis from bile duct malignancy. This case is notable for atypical presenting features, including a trunk eruption comprised of excoriated papules with onset following meloxicam initiation, mimicking a cutaneous adverse drug reaction. Providers should consider systemic etiologies of pruritus in patients presenting with cutaneous eruptions with atypical features. Accurate categorization of pruritus can facilitate treatment and/or additional investigation of systemic disease.

## Introduction

Pruritus is a frequent symptom in patients with cholestatic liver disease. Clinically, cholestatic pruritus may be generalized or localized to the limbs, primarily the palms and soles. Classical symptoms associated with cholestatic pruritus include jaundice and/or scleral icterus, dark urine, light-colored stools, abdominal pain, nausea, and vomiting [1,2]. Here, we present a case of cholestatic pruritus secondary to bile duct malignancy mimicking a cutaneous adverse drug reaction. Our case highlights the importance of screening for systemic disease underlying pruritus with atypical cutaneous eruptions.

## Case presentation

An 83-year-old male presented to the outpatient dermatology clinic with a 10-day history of generalized pruritus, intermittent low-grade fever, and truncal rash. The patient reported starting meloxicam three weeks earlier for a right finger avulsion fracture followed by symptoms of dark urine and pale stools. Meloxicam was discontinued after seven days of use with resolution of urinary and stool symptoms; however, days later, the patient developed significant generalized pruritus with a truncal rash that his primary care provider (PCP) understandably felt was most likely a potential drug reaction to meloxicam. He was prescribed topical hydrocortisone and seen in the dermatology clinic four days later. Topical hydrocortisone provided some improvement but the pruritus remained persistent. His past medical history was significant for a 60-pack-year smoking history with cessation seven years prior, chronic obstructive pulmonary disease, essential hypertension, and chronic kidney disease.

**Figure 1:**
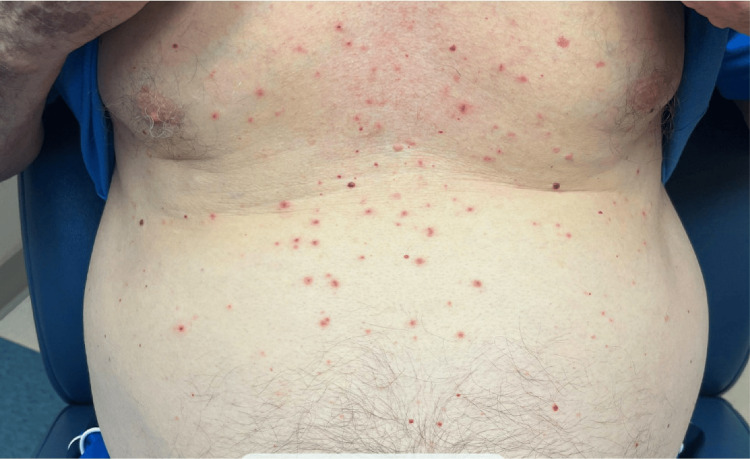
Clinical presentation of maculopapular eruption with pink papules with central crusting predominantly on the chest and abdominal area

Physical exam was notable for an eruption consisting of pink papules with central crusting predominantly on the abdomen and chest with few scattered lesions on the upper and distal lower extremities (Figure 1). No jaundice or scleral icterus was observed.

The appearance of the rash was atypical for a drug reaction, with localized involvement and primarily secondary changes with excoriated papules. Topical triamcinolone and oral hydroxyzine were prescribed to manage pruritus. A complete blood count, comprehensive metabolic panel, and urinalysis were ordered. Labs were obtained the same day and were significant for elevated bilirubin (7.8 mg/dL), elevated alkaline phosphatase (532 IU/L), elevated aspartate aminotransferase (160 IU/L), and elevated alanine transaminase (210 IU/L) (Table 1).

**Table 1:** Laboratory values WBC: white blood cells; RBC: red blood cells; MCV: mean corpuscular volume; BUN: blood urea nitrogen; AST: aspartate aminotransferase; ALT: alanine transaminase.

Laboratory value	Reference range	Patient result
WBC	4.8-10.8 x 10^9^/L	9.2
RBC	4.7-6.1 x 10^12^/L	4.36
Hemoglobin	14.0-18.0 g/dL	13.7
Hematocrit	42.0-52.0%	40.8
MCV	80.0-94.0 fL	93.6
Platelet count	150-450 x 10^9^/L	324
Glucose	70-100 mg/dL	129
BUN	8-27 mg/dL	32
Creatinine	0.6-1.6 mg/dL	1.53
Sodium	136-145 meq/L	142
Potassium	3.5-5.3 meq/L	4.4
Chloride	97-111 meq/L	110
Bilirubin (total)	0.2-1.2 mg/dL	7.8
Alkaline phosphatase	32-130 IU/L	532
AST	0-40 IU/L	160
ALT	0-68 IU/L	210
Total protein	6.0-8.0 g/dL	7.7
Albumin	3.2-4.6 g/dL	3.7

**Figure 2:**
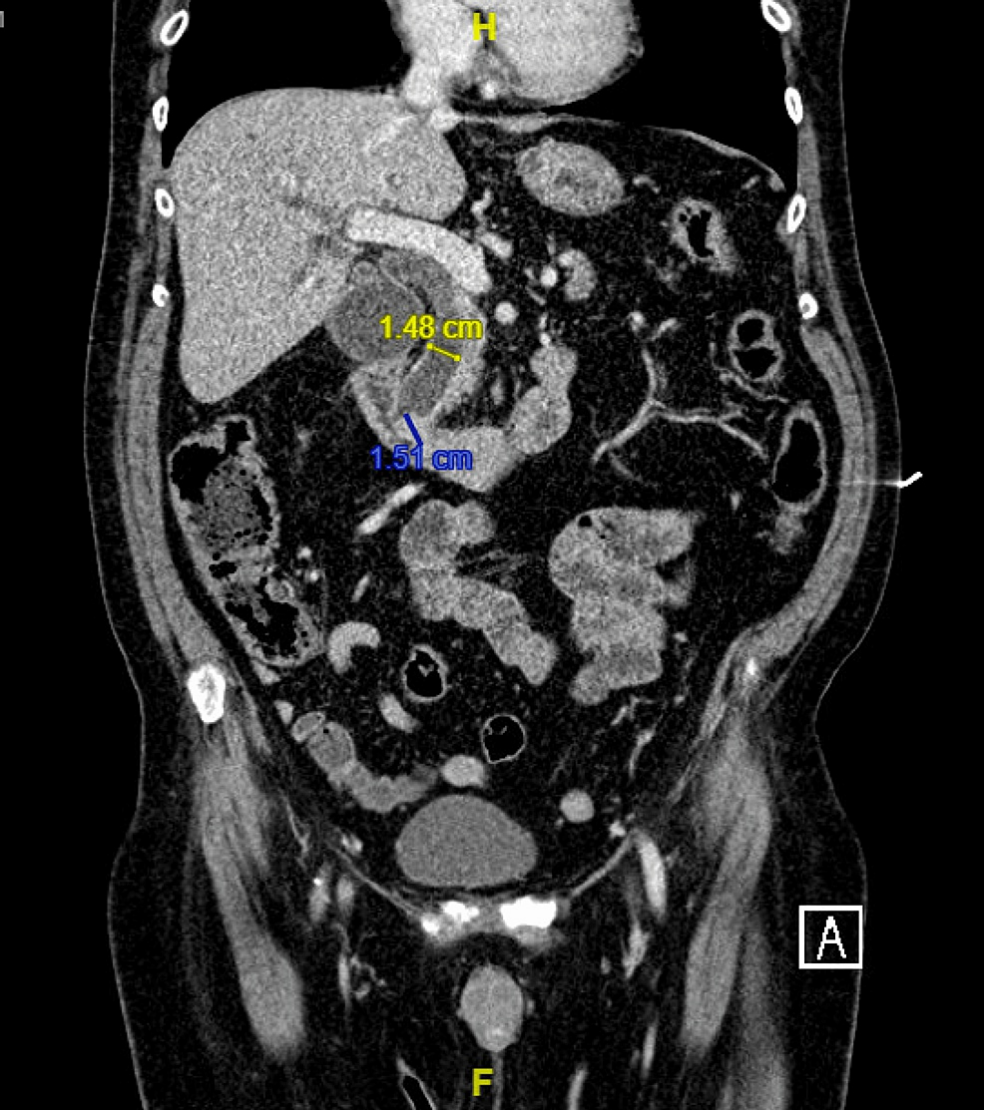
CT scan of abdomen and pelvis significant for 1.8 x 1.3 x 1.5 cm mass within the common bile duct resulting in marked bile duct dilatation

**Figure 3:**
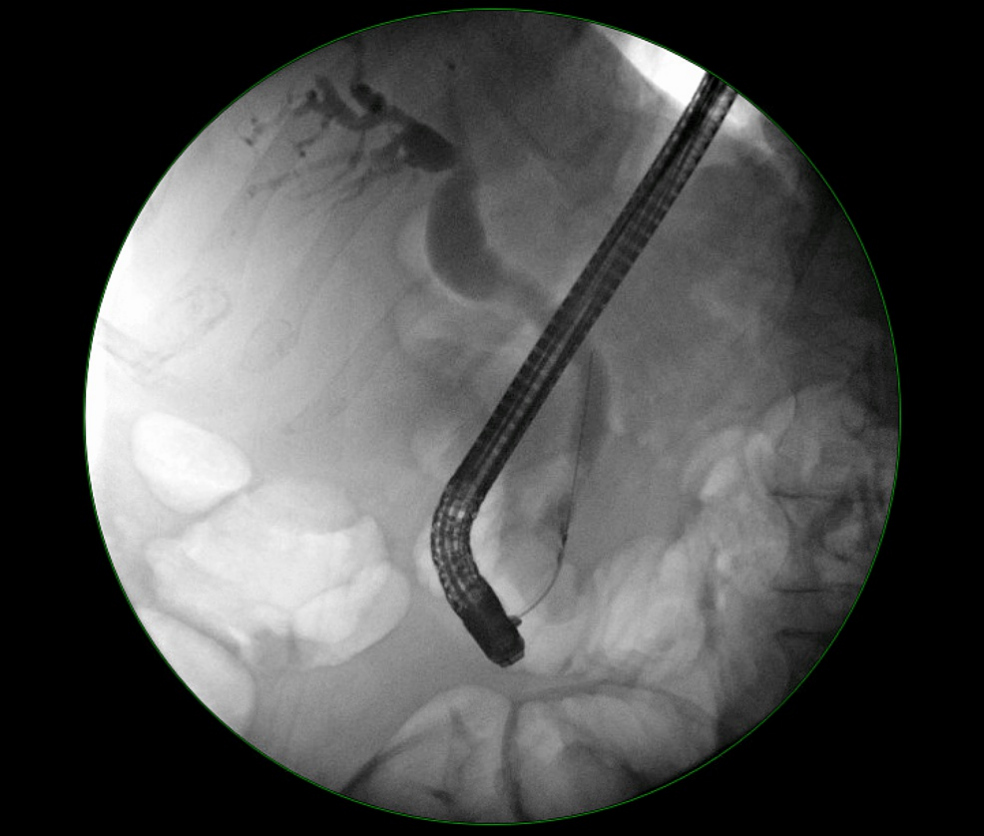
Endoscopic retrograde cholangiopancreatography suggestive of potential distal bile duct cholangiocarcinoma or ampullary mass invading into distal bile duct

The patient was sent to the emergency room, where a computed tomography (CT) scan of the abdomen and pelvis was significant for a 1.8 cm mass within the common bile duct (Figure 2). Subsequent endoscopic retrograde cholangiopancreatography (ERCP) was suggestive of potential distal bile duct cholangiocarcinoma or ampullary mass invading into distal bile duct (Figure 3). Sphincterotomy and stent placement for drainage were performed, and a distal bile duct brushing and ampullary biopsy were also obtained. The next day, bilirubin had decreased to 4.8 mg/dL and the patient reported symptomatic improvement in pruritus. The patient was discharged, with outpatient follow-up with gastroenterology arranged for further management. Cytology results from the distal bile duct brushing indicated atypical cells suspicious for carcinoma, but results from the ampullary biopsy were inconclusive, noting areas of atypia but no definite carcinoma in the sampled area. However, based on the patient's presentation and endoscopic impression of the mass lesion, a clinical diagnosis of cholangiocarcinoma was made and surgical removal of the biliary mass with a Whipple procedure was performed.

## Discussion

Pruritus, or itch, is a common clinical symptom that is coded in approximately 1% of all ambulatory care visits annually in the United States [3]. The etiology of the underlying pruritus varies but can broadly be classified into four categories: pruritoceptive, neuropathic, neurogenic/systemic, and psychogenic [4-6].

Pruritoceptive itch is dermatological in origin and arises due to inflammation, dryness, or other cutaneous damage, which often manifests with primary lesions [4,5]. Pruritoceptive pruritus is seen in common dermatological conditions, including xerosis, urticaria, and dermatitis. Approximately 50% of patients with primary dermatological diagnoses report pruritus as an accompanying symptom [6]. It is primarily transmitted by demyelinated type C and thinly myelinated type A-delta nerve fibers in the skin [7].

In contrast to pruritoceptive pruritus, neurogenic or systemic pruritus originates from noncutaneous organ systems and is transmitted from the central nervous system without peripheral nerve involvement. Systemic pruritus is associated with underlying renal, hepatobiliary, hematologic, oncologic, rheumatological, or endocrine disease. Common associated diagnoses include cholestasis, chronic kidney disease, myeloproliferative disorders, lymphoma, and thyroid disease [4-6]. Neuropathic pruritus develops due to excess activity of peripheral nerves or decreased inhibition of central neurons in the itch pathway, while psychogenic pruritus is seen in patients with underlying anxiety, depression, or other psychiatric conditions [4,8].

Accurate categorization of pruritus can facilitate treatment and further work-up. In particular, appropriate consideration of systemic etiologies of pruritus can be key in diagnosing underlying disease. In the reported case, the patient presented with systemic pruritus secondary to cholestasis from bile duct malignancy. Pruritus is a frequent symptom in patients with cholestatic liver disease. The exact pathogenesis of pruritus in cholestasis remains unclear but is theorized to involve multiple pruritogens, including bile acids, endogenous opioids, and lysophosphatidic acid [1]. The prevalence of pruritus varies depending on the underlying liver disease, ranging from 45% of patients with malignant biliary obstruction to 16% of patients with benign obstructions [1]. Clinically, cholestatic pruritus may be generalized or localized to the limbs, primarily the palms and soles. Classical symptoms associated with cholestatic pruritus include jaundice and/or scleral icterus, dark urine, light-colored stools, abdominal pain, nausea, and vomiting [1,2].

Systemic causes of pruritus such as cholestasis are not typically associated with primary skin lesions, which are more suggestive of a pruritoceptive etiology of pruritus. However, intense scratching from pruritus may result in secondary lesions due to excoriation, lichenification, prurigo nodularis, or other disturbances that may resemble primary skin lesions [2]. In this case, the area of skin involvement despite the diffuse pruritus reported is reflective of the patient’s ability to physically reach areas such as the abdomen and to a lesser degree, arms and legs, compared to the back where no lesions were present.

Additional atypical features of this case include the absence of jaundice or scleral icterus often seen in cholestatic pruritus, as well as the absence of abdominal pain, nausea, or vomiting [2]. The classical symptoms of dark urine and light-colored stools were reported; however, the onset and resolution of urinary and stool symptoms correlated with meloxicam initiation and cessation suggesting drug-induced etiology.

Patients with persistent pruritus without primary skin lesions should be considered candidates for systemic pruritis screening. However, it should be recognized that skin lesions secondary to excoriation may be indistinguishable from primary skin lesions based solely on physical exam, and preliminary screening despite the atypical cutaneous presentations may be required. Initial screening for systemic pruritus can include a complete blood count with differential, comprehensive metabolic panel, glycosylated hemoglobin, and additional hepatic, renal, and thyroid function testing as needed [5,6].

## Conclusions

Pruritus is a common clinical symptom seen in a broad range of dermatologic and systemic disorders. This case highlights the importance of screening for systemic causes of pruritus in all patients presenting with persistent pruritus with atypical features. Although the patient in the presented case had few signs of systemic disease and atypical cutaneous lesions, screening laboratory work revealed underlying cholestasis and prompted immediate intervention. Appropriate screening in patients with pruritus can facilitate timely diagnosis and treatment of underlying disease.
